# Corneal cross-linking versus standard care in children with keratoconus – a randomised, multicentre, observer-masked trial of efficacy and safety (KERALINK): a statistical analysis plan

**DOI:** 10.1186/s13063-020-04392-1

**Published:** 2020-06-12

**Authors:** Kashfia Chowdhury, Caroline J. Doré, Catey Bunce, Daniel F. P. Larkin

**Affiliations:** 1 Comprehensive Clinical Trials Unit, Institute of Clinical Trials and Methodology, University College London, London, UK; 2 School of Population Health and Environmental Sciences, Faculty of Life Sciences and Medicine, King’s College London, London, UK; 3 NIHR Moorfields Clinical Research Facility, Moorfields Eye Hospital, London, UK

**Keywords:** Statistical analysis plan, Keratoconus, Cross-linking, Surgery, Randomised controlled trial, Pentacam topography, K_2_, Paediatric

## Abstract

**Background:**

The KERALINK trial tests the hypothesis that corneal cross-linking (CXL) treatment reduces the progression of keratoconus in comparison to standard care in patients aged 10–16 years. This article describes the statistical analysis plan for this trial as an update to the published protocol. It is written before the end of the patient follow-up, while the outcome of the trial is still unknown.

**Design and methods:**

KERALINK is a randomised controlled, observer-masked, multicentre trial in progressive keratoconus comparing epithelium-off CXL with standard care, including spectacles or contact lenses as necessary for best-corrected acuity. Keratoconus is a disorder of the shape of the cornea in which the normally round dome-shaped clear front window of the eye (cornea) thins progressively leading to a cone-like bulge. This impairs the ability of the eye to focus properly, causing reduced vision which requires spectacle or contact lens wear or, in a minority of patients, eventually corneal replacement by a transplant for best vision. The primary outcome measure is the between-group difference in K_2_ at 18 months adjusted for K_2_ at baseline examination. K_2_ is the value of the steepest corneal meridian as measured on Pentacam topography. Secondary outcomes are keratoconus progression, time to keratoconus progression, visual acuity, refraction, apical corneal thickness and adverse events. Patient-reported effects will be explored by questionnaires. We describe in detail the statistical aspects of KERALINK: the outcome measures, the sample size calculation, general analysis principles, the planned descriptive statistics and statistical models, and planned subgroup and sensitivity analyses.

**Discussion:**

The KERALINK statistical analysis will provide comprehensive and precise information on the relative effectiveness of the two treatments. The plan will be implemented in May 2020 when follow-up for the trial is completed.

**Trial registration:**

EudraCT, 2016-001460-11. Registered on 19 May 2016.

## Background

Keratoconus is characterised by thinning and distortion of the cornea that results in visual loss from complex refractive error and corneal opacification. The prevalence in Europe is 1:12,000, rising to 1 in 450 in South Asians, with an estimated 50,000 affected individuals in the UK.^1^ Keratoconus is often more advanced if it is first diagnosed in childhood, with faster subsequent disease progression. In its early stages, keratoconus causes worsening of vision: spectacle correction provides good visual acuity in early disease only, until increasingly irregular astigmatism requires correction with rigid contact lenses for best vision. Lenses may not be well tolerated for significant periods of the day because of the irregular shape of the cornea and the common association of keratoconus with severe allergic eye disease. Without lenses these individuals can effectively be blind.

Cross-linking (CXL) is a procedure conceived to increase the stiffness of the cornea and stop progression of keratoconus. Epithelium-off cross-linking is a procedure that involves surgical removal of the outer layer of the cornea before administration of riboflavin eye drops and exposure of the cornea to ultraviolet (UV) light. The KERALINK prospective, multicentre, parallel-group, observer-masked randomised controlled trial (RCT) will investigate the efficacy and safety of the established epithelium-off CXL in the paediatric age group, in which no RCT has been undertaken. Full details of the background to the trial, the interventions under study and its design are in the published trial protocol [1].

This article describes the statistical analysis plan (SAP) for the KERALINK trial. Analyses will begin in May 2020 after completion of the 18-month follow-up for the last patient, data cleaning checks and data lock. However, if the last patient is observed to have progression in the study eye at the 18-month visit, a further confirmatory visit will take place in July 2020 and analysis will begin soon after. The analysis of the primary outcome will be independently programmed from the cleaned derived dataset by a statistician who did not perform the main analysis, and in parallel by the trial statistician.

### Objectives

The primary objective of the KERALINK trial is to compare CXL versus standard care at 18 months from randomisation and to investigate whether CXL is efficacious in stabilising the progression of keratoconus and safe in children and young people aged 10–16 years.

## Design and methods

### Design

KERALINK is a two-arm, prospective, multicentre, parallel-group, observer masked RCT.

### Patient eligibility criteria

#### Inclusion criteria

- Age 10–16 years with keratoconus progression confirmed in one or both eyes using Pentacam or other topography devices. Progression for eligibility is defined as an increase of at least 1.5 D in K_2_ or K_max_ on Pentacam corneal topography (or equivalent on other topography devices) between two examinations done using the same scanning technique at least 3 months apart;
- Patients and their parents/guardians must be sufficiently fluent in English to provide assent and informed consent and to complete the patient-reported outcome measures;
- Patients must be willing to attend for follow-up visits.

#### Exclusion criteria

- Advanced keratoconus as determined by apex corneal scarring;
- Apex corneal thickness < 400 μ;
- Steepest corneal meridian (K_2_) > 62 D and maximum corneal curvature (K_max_) > 70 D on Pentacam topography at screening;
- Rigid contact lens wear in both eyes and unable to abstain for 7 days pre-examinations;
- Corneal co-morbidity;
- Down’s syndrome;
- Any clinical condition which the investigator considers would make the patient unsuitable for the trial, including pregnancy;
- Participation in other clinical trials which would materially impact on the KERALINK study.

### Randomisation and blinding

The randomisation process was based on a minimisation algorithm which incorporates a random element with minimisation factors of treatment centre and whether progression is confirmed in one eye or both eyes at randomisation. An independent online randomisation service (Sealed Envelope) conducted the randomisation to minimise allocation bias within the trial.

Patients were allocated in a 1:1 ratio to the CXL and standard care arms. Due to the nature of the intervention, neither the trial participants nor the treating clinicians or site staff were masked to the treatment allocation. However, optometrists performing outcome assessments were unaware of treatment allocation. The Principal Investigator (PI) or treating clinicians were not aware of the primary outcome values measured during the follow-up assessments.

### Trial intervention

At randomisation, patients were allocated to receive either CXL or standard care.

For CXL, surgery is performed under general or local anaesthesia as applicable, followed by standard management. After removal of the corneal epithelium and administration of riboflavin drops, UV light is administered according to standardised parameters of 10 mW/cm^2^ for a 5.4 J/cm^2^ total energy dose.

Standard care includes refraction testing with the provision of glasses and/or specialist contact lens fitting. Glasses or contact lenses are to be provided for one or both eyes as required for best corrected visual acuity. Those patients who develop advanced disease and poor spectacle- and lens-corrected visual acuity during the course of the trial will be offered corneal transplantation. Full details of these interventions can be found in the trial protocol [1].

## Outcomes

### Primary outcome measure

The primary outcome measure is the difference between the two arms in K_2_ in the study eye at 18 months post-randomisation using standard Pentacam imaging. K is the corneal power at a given point on Pentacam topography, measured in D. K_2_ is the value of the steepest corneal meridian and more representative than K_max_, which is the corneal power at the steepest point in the cornea. For each patient, the eye with the more advanced keratoconus (highest value of K_2_ and with documented increase of > 1.5 D between examinations before randomisation) at the time of randomisation will be defined as the study eye for the primary analysis, unless that eye has previously been treated by CXL or corneal transplantation. The 18-month power will be used if it is taken in a window of ±28 days.

### Secondary outcome measures

The secondary outcome measures for the trial are:

- Keratoconus progression (yes/no) defined as > 1.5 D increase in K_2_ from baseline (at randomisation) to 18 months after randomisation or requirement for change from spectacle to rigid contact lenses correction of vision, as the latter precludes reliable topography measures;
- Time from randomisation to keratoconus progression (defined as > 1.5 D increase in K_2_ from baseline);
- Uncorrected and best corrected visual acuity (measured as logMAR using EDTRS chart);
- Refraction (measured dioptres spherical equivalent, myopia and astigmatism);
- Apical corneal thickness measurement (ultrasound);
- Quality of life as assessed by CHU9D and CVAQC questionnaires.

### Details of outcome measures

#### K_2_ measurement

The K_2_ measurements from Pentacam images will be used as the indicator of disease progression. The probability is high that increases of > 1.5 D in K_2_ would discriminate a true change in the steepest corneal meridian from artefact. A change of this magnitude is clinically significant, indicating a likelihood of improved visual acuity with correction of the refractive change; for example, benefit from spectacle provision in an eye that previously had good unaided vision, a change in spectacle lens correction or progression from spectacle wear to contact lens correction. K_2_ will be measured three times by a masked observer at each trial visit and the mean value used in analyses.

Taking into account the likelihood of inter- and intra-test variation in topography analysis, any patient found to have > 1.5 D increase in K_2_ from randomisation will need to have this confirmed at a subsequent visit (i.e. 3 months later). Participants who have unconfirmed progression at the 18-month follow-up visit will need this confirmed at a further visit at 21 months.

#### Uncorrected and best corrected visual acuity (measured as logMAR using EDTRS chart)

Distance visual acuity (DVA) is recorded as the number of correct letters read in the ETDRS chart at a distance of 4 m. The ETDRS chart comprises 14 lines with five letters per line (i.e. 70 letters in total). With the ETDRS scoring system:

- If ≥ 20 letters are read correctly at a starting distance of 4 m, the visual acuity score is equal to the number of letters read correctly + 30.
- If < 20 letters are read correctly at a starting distance of 4 m, the visual acuity score is equal to the number of letters read correctly at 4 m plus the number of letters read correctly at 1 m in the first six lines.
- If no letters are read correctly at either the 4-m distance or the 1-m distance, tests counting fingers (CF), hand motion (HM), perception of light (PL) and no perception of light (NPL) will be performed.

The visual acuity score will be converted to logMAR equivalents using the formula:

$$ logMAR=1.7-0.02\times \left( Visual\ acuity\ score\right) $$

For patients that cannot read any letters correctly in the EDTRS chart at a distance of 1 m, assessments of CF, HM, PL and NPL will be assigned VA logMAR values of 2.10, 2.40, 2.70 and 3.00, respectively. Therefore, VA logMAR will be in the range of − 0.3 to 3.0 with lower values indicating better vision.

The uncorrected versus corrected refers to whether the person has their vision assessed with glasses (or lenses) or without.

#### Refraction

Refractive data will be summarised in two different ways for analysis: (1) as the spherical equivalent refractive error (SER); and (2) as the compound spherocylinder.

1. The SER will be calculated by adding half of the cylinder power (cyl) to the sphere power:
  $$ SER= sphere+\left(\frac{\  cyl}{2}\right) $$

The number of patients with refractive astigmatism will be based on refractive cylinder. An absolute cyl of 0.75D or more represents significant refractive astigmatism.

(2) Mean refraction of compound spherocylinder will be obtained at baseline and at 18 months after randomisation by converting the data into matrix form. We will use Keating’s method based on Long’s dioptric power matrix to obtain the mean refractive treatment effect from pre-randomisation and 18-month refraction data. The matrices of refractive treatment effects in each arm will be used to test for statistical significance of a mean difference between the arms [2]. The multivariate test statistic, w, will be used to determine whether the difference between the two means is significant.

$$ w={\overline{\mathrm{a}}}^{\prime }{{\mathrm{S}}_{\mathrm{a}}}^{-1}\overline{\mathrm{a}}N\left(N-3\right)/3\left(N-1\right) $$

Where $\bar {\mathrm{a}}$ is the coordinate vector of mean difference in dioptric power matrices of refractive treatment effects, and S_a_ is the variance-covariance matrix for $\bar {\mathrm{a}}$. The test statistic will be compared to the F-distribution with 3 and N-3 degrees of freedom.

#### Apical corneal thickness measurement

Biomechanical and ultrastructural studies to date have not been able to demonstrate the mechanism by which CXL stiffens the cornea. The KERALINK study will examine changes in thickness of the cornea using ultrasound as topography measurements do not provide accurate and reproducible thickness measurements.

#### The Child Health Utility 9D (CHU9D)

The CHU9D is a paediatric generic preference-based measure of health-related quality of life. It consists of a descriptive system and a set of preference weights, giving utility values for each health state described by the descriptive system, allowing the calculation of quality adjusted life years (QALYs) for use in cost utility analysis. It has been validated for self-completion in an adolescent population (11–17 years) [3–7]. The questionnaire consists of nine items, each with a five-level response category. Each item relates to a particular domain: worry; sadness; pain; tiredness; annoyance; school; sleep; daily routine; and activities.

#### CVAQC

This is a 25-item vision specific questionnaire designed for children [8]. The answers, selected on a 4-point scale, cover areas such as education, near and distance vision, getting around, social interaction, entertainment and sports. The raw CVAQC scores will be transformed into logarithmic scores using a Rasch calculator designed by the developers of the questionnaire. Questionnaires are invalid if they have > 33% missing data.

## Sample size

A difference between the groups in the change in K_2_ of 1.5 D from randomisation to 18 months would be viewed as a clinically important difference (based on Wittig-Silva RCT of CXL in adults [9]). A K_2_ increase > 1.5 D would discriminate a true change in the steepest corneal meridian from measurement artefact and would be visually significant.

A sample size of 46 patients would be required to detect this difference at the 5% significance level with 90% power, assuming a SD of 1.5 D. The total sample size has been increased to 60 patients (30 per group) to allow for up to 24% loss to follow-up. These estimates are based on 12- and 24-month data reported by Wittig-Silva et al., from which we estimate a pooled SD of the changes of 1.476 D.

We expect that, on average, there will be a 10% loss to follow-up in both groups. In the study by Wittig-Siva et al., 19% of patients withdrew, crossed over to CXL or had a transplant by 18 months. However, 18% of patients in the control group received either CXL or a transplant. If we specifically adjust the sample size to take account of 10% loss to follow-up and up to 20% of the control arm cross-over to CXL or transplant, then our planned total sample size of 60 patients would still provide at least 80% power to detect the clinically important difference. The trial design dictates that children cannot cross over to CXL before 9 months from randomisation.

## Analysis principles

### Adherence to protocol

Adherence to the protocol requires that: (1) the patient takes the treatment they were randomised to; (2) the patient does not cross-over to CXL before 9 months after randomisation; and (3) the patient attends all visits within the visit window as per the protocol. Patients are encouraged to continue providing follow-up measurements, even if they stop adhering to the protocol before 18 months.

### Patient population to be included in analysis

The main analysis will be conducted on an intention-to-treat (ITT) basis; all observed outcome data from randomised patients according to their allocated treatment arm will be used, irrespective of whether they receive the randomised treatment. Sensitivity analysis (described below) will assess the impact of missing outcome data.

Additionally, if cross-over does occur, a per-protocol (PP) analysis will be performed for the primary outcome that only includes data from patients before crossing over. This analysis will exclude data for patients who do not receive the treatment they were randomised to.

### Significance levels of tests and confidence intervals

All statistical tests will use a two-sided *p* value of 0.05, unless otherwise specified. There will be no formal adjustment of *p* values. Two-sided 95% confidence intervals will be presented for all estimates.

### Baseline comparability

Baseline characteristics will be summarised by randomised treatment arm. Categorical variables will be summarised by number and percentage in each category; continuous variables will be summarised by mean and standard deviation or median and interquartile range, as appropriate. No statistical tests of differences in baseline characteristics between groups will be done, as any differences between treatment arms must be due to chance.

### Adjustment for design factors

Since randomisation is stratified by treatment centre and number of eyes progressed at randomisation, analyses of outcomes will involve adjustment for these factors (as recommended in ICH E9, section 5.7 [10]) unless otherwise indicated. Treatment effects will then be estimated conditional on treatment centre and whether one or both eyes progressed at randomisation.

Baseline K_2_ will also be adjusted for in primary analyses where this is the outcome. Similar adjustment will be made for all continuous secondary outcome variables where a baseline measurement is recorded.

### Follow-up and losses to follow-up: missing data

Missing baseline covariate data are not anticipated since covariates must be recorded to allocate treatment.

We expect that up to 10% of patients will not provide measurements at the 18-month follow-up. Numbers and percentages of missing data at each visit (at baseline and 18 months) will be tabulated by treatment group for the primary and secondary outcomes.

All observed data will be included in the primary and secondary analyses. Missing outcome data will be assumed to be missing-at-random (MAR) conditional on the observed values of all other variables included in the analysis models, and so independent of the values of the unobserved data itself. As the primary outcome model is adjusted for baseline K_2_, patients without baseline and at least one outcome score will consequently not be included in the analysis. Their inclusion, however, would not add any information to the analysis [11].

## Statistical analyses

All analyses will be carried out using Stata version 15 (or above). The results of the analyses will be reported following the principle of the ICH E3 guidelines on the Structure and Content of Clinical Study Reports [12] and CONSORT guidelines [13].

### Recruitment and follow-up patterns

The number of patients screened for eligibility will be presented. Reasons for non-admission into the trial will be reported.

The period of data collection, including the date of the first patients’ first visit and date of the last patients’ last visit will be described. Recruitment will be presented by year and centre. The throughput of patients from those screened, those randomised and those assessed at each visit and included in the analysis will be summarised in a CONSORT flowchart [13]. The number of patients who withdraw and are unwilling to provide follow-up will be reported by treatment arm. The number of cross-over or treatment deviations will be reported by treatment arm.

### Baseline characteristics

Baseline characteristics will be summarised in a table by treatment arm. The variables to be reported in the baseline tables are listed in the dummy tables (Additional File 1, Table A1).

### Trial treatment

The number of patients undergoing their randomised surgery will be reported by treatment group. There is provision for patients to cross-over from one arm to another in this trial. However, the trial design dictates that children cannot cross-over to CXL before 9 months. Any cross-overs or other treatment deviations, such as visits outside treatment window, as well as the number of patients who did not receive the allocated treatment will be specified along with reasons, as detailed in the protocol deviation log.

### Analysis methods

#### Primary analysis

A multilevel repeated measures linear regression model will be used to estimate the difference between the treatment groups in K_2_ values at 18 months after randomisation. This analysis model will use all available visit data (from 3 months to 18 months) to strengthen confidence in the MAR assumption and provide greater power to detect a difference at the 18-month visit and test for superiority.

The model will include fixed effects for K_2_ at randomisation (continuous), treatment group (two categories: CXL and Standard care), time (six categories: 3, 6, 9, 12, 15 and 18 months), and the minimisation factors centre and number of eyes progressed at randomisation. A random patient effect will be included to take account of clustering by patient. The model for K_2_, where y_ij_ is the K_2_ of patient i at time j, is:

$$ {\mathrm{y}}_{\mathrm{i}\mathrm{j}}={\beta}_{\mathrm{i}0}+{\beta}_1\left({\mathrm{Y}}_{\mathrm{i}0}\right)+{\beta}_2\left({\mathrm{treatment}}_{\mathrm{i}}\right)+{\beta}_3\left({\mathrm{time}}_{\mathrm{j}}\right)+{\beta}_4\left({\mathrm{time}}_{\mathrm{j}}\ast {\mathrm{treatment}}_{\mathrm{i}}\right)+{\beta}_5\left({\mathrm{centre}}_{\mathrm{i}}\right)+{\beta}_6\left({\mathrm{eyes}}_{\mathrm{i}}\right) $$

Where, *β*_i0_ = *β*_0 +_*u*_i0_ + ε_ij_, ${u}_{\mathrm{i}0}\sim \mathrm{N}\Big(0,{\sigma}_{u0}^2$), ε_ij_ ~ N(0,*σ*^2^) and treatment = 1 if CXL and 0 if Standard care.

The primary outcome is the average difference between treatment groups at 18 months, estimated as *β*_2_ + *β*_4_(*time* = 18 *months*).

The model coefficients will be estimated using robust standard errors, to allow for the possibility of unequal variances in the two randomised groups.

The model makes assumptions about random effects distributions, correlation structure and residuals, which will all need investigation. If any assumptions are poorly met, then transformation of the K_2_ measures may be required.

Model assumptions will be assessed and a logarithmic transformation may be used if this improves normality of the residuals.

### Secondary analysis

#### Continuous secondary outcomes

Each of the following continuous secondary outcome measures on the study eye will be analysed using a separate multilevel repeated measures linear regression model:

- Uncorrected and best corrected visual acuity (measured as logMAR using EDTRS chart);
- Apical corneal thickness measurement (ultrasound);
- Spherical equivalent refraction (SER).

In patients for whom both eyes show progression at the time of randomisation, information from both eyes will be included in a secondary analysis of the continuous secondary outcomes. Each model will include fixed effects for treatment, time, treatment by time, baseline value of the associated outcome and the stratifying variables. A random patient effect will be included to take account of clustering by patient.

The mean refractive surgical effect (RSE) will be calculated from pre-randomisation and 18-month refraction data using matrices. We will use the appropriate test for dioptric power to compare RSE between arms.

#### Categorical secondary outcomes

Logistic regression models will be fitted to estimate the effect of treatment for each categorical variable:

- Keratoconus progression (yes/no) is defined as > 1.5 D increase in K_2_ from randomisation to 18 months or requirement for change from spectacle to rigid contact lenses for correction of vision, as the latter precludes reliable topography measures. Acknowledging inter- and intra-test variation in topography analysis, any patient found to have > 1.5 D increase in K_2_ will need to have progression confirmed at a subsequent visit (i.e. 3 months later);
- Refractive astigmatism.

#### Time-to-event outcome

Time to keratoconus progression will be visually displayed using Kaplan–Meier curves; the difference between the arms in the time from randomisation to progression will be compared using Cox regression models which include the minimisation factors. The first date when progression is observed will be the date used for this analysis.

#### Adverse events and serious adverse events

The proportion of patients experiencing at least one adverse event (AE) and those experiencing at least one serious adverse event (SAE) will be summarised by treatment arm. The number and percentage of AEs and SAEs will be presented descriptively, but no formal analysis will be performed.

The proportion of patients with any SAE or AE in the study eye will be compared between the two arms.

### Additional analyses

#### Subgroup analyses

The regression model for the primary outcome will be extended by adding interaction terms to investigate the effect of treatment on pre-specified subgroups. We will include interactions between treatment and number of eyes progressed at randomisation, ethnicity and family history of keratoconus/atopy to investigate whether the effect of treatment differs by each of these factors. As the trial has not been powered to detect this, the analysis will have limited power and is exploratory. We will also use forest plots to graphically display treatment effects across the subgroups. All subgroup analyses are hypothesis generating and will not form the basis of conclusions drawn from the trial.

#### Exploratory outcome analysis

The steepest corneal curvature measured by Pentacam, K_max_, will be analysed as an exploratory outcome, using a similar model as the primary outcome analysis.

We will carry out exploratory analyses using K_2_ and K_max_ values to identify which is a more appropriate measure of clinically/visually significant progression of keratoconus.

#### Sensitivity analyses

Sensitivity analyses will be conducted on the primary outcome to assess the robustness of results to treatment cross-over or failure to receive any treatment following randomisation. In the event of cross-over from one randomised arm to the other, we will perform analyses of the primary outcome on a per-protocol basis. The per-protocol analysis will exclude any information collected from a patient after cross-over. This analysis will also exclude data for patients who did not receive the treatment to which they were randomised. Any cross-over or treatment deviations will be summarised with reasons.

## Discussion

This update contains the pre-specified SAP for the KERALINK trial. By publishing the SAP, we aim to increase the transparency of the data analysis. The KERALINK trial will provide comprehensive and precise information on the relative effectiveness of cross-linking versus standard care in children with keratoconus.

## Supplementary information

**Additional file 1.** Dummy tables. This file contains dummy tables which show the planned format and contents of the tables for the KERALINK final statistical report.

**Additional file 2.** Guidelines for the Content of Statistical Analysis Plans in Clinical Trials Checklist (1) (recommended by the EQUATOR Network).

## Data Availability

The protocol has previously been published [1]. After completion of the trial analysis, the results will be published and additional available data can be obtained by contacting the Chief Investigator (DFPL). The study team retain exclusive use until publication of major outputs has been completed.

## Abbreviations

AE: Adverse event
AR: Adverse reaction
CCTU: Comprehensive Clinical Trials Unit
CI: Chief Investigator
CRF: Case report form
CTA: Clinical trial authorisation
CXL: Corneal cross-linking
DSUR: Development Safety Update Report
EU: European Union
FDA: (U.S.) Food and Drug Administration
FWA: Federal Wide Assurance
GCP: Good Clinical Practice
ICH: International Conference on Harmonisation
IMP: Investigational Medicinal Product
IRB: Institutional Review Board
ITT: Intention to treat
K_2_: The steepest corneal meridian in dioptres on Pentacam topography
K_max_: Maximum corneal curvature measured on corneal topography
MHRA: Medicines and Healthcare products Regulatory Agency
PI: Principal Investigator
PIS: Participant Information Sheet
QA: Quality assurance
QC: Quality control
QoL: Quality of life
QMMP: Quality Management and Monitoring Plan
R&D: Research and Development
REC: Research Ethics Committee
SAE: Serious adverse event
SAP: Statistical analysis plan
SAR: Serious adverse reaction
SPC: Summary of product characteristics
SSA: Site-specific approval
SUSAR: Suspected unexpected serious adverse reaction
TMF: Trial master file
TMG: Trial Management Group
TMT: Trial Management Team
ToR: Terms of feference
TSC: Trial Steering Committee
UCL: University College London
VA: Visual acuity
