# Supplementary material for: Corneal cross-linking versus standard care in children with keratoconus – a randomised, multicentre, observer-masked trial of efficacy and safety (KERALINK): a statistical analysis plan
Source: Trials. 2020 Jun 12;21:523. doi: 10.1186/s13063-020-04392-1 (PMC7291687; doi:10.1186/s13063-020-04392-1)
Supplement: Supplementary file 1 — Additional file 1. Dummy tables. This file contains dummy tables which show the planned format and contents of the tables for the KERALINK final statistical report. [file 13063_2020_4392_MOESM1_ESM.docx]

# Additional File 1 – Dummy Tables

## Table A1. Baseline Characteristics

| **Stratification factors** |  | **CXL** | **Standard care** | **Total** |
| --- | --- | --- | --- | --- |
|  |  | n= | n= | N= |
| Treatment centre | n (%) |  |  |  |
| Moorfields |  |  |  |  |
| Sheffield |  |  |  |  |
| Liverpool |  |  |  |  |
| Newport |  |  |  |  |
| Manchester |  |  |  |  |
| Number of eyes progressed and eligible | n (%) |  |  |  |
| One eye |  |  |  |  |
| Both eyes |  |  |  |  |
| **Characteristic at screening** |  |  |  |  |
| Age (years) | mean(sd) |  |  |  |
| Gender | n(%) |  |  |  |
| Female |  |  |  |  |
| Male |  |  |  |  |
| Ethnicity | n(%) |  |  |  |
| White |  |  |  |  |
| Mixed |  |  |  |  |
| Black or Black British |  |  |  |  |
| Asian or Asian British |  |  |  |  |
| Other Ethnic groups |  |  |  |  |
| Refractive corrective aid – *Study eye* | n(%) |  |  |  |
| Glasses |  |  |  |  |
| Soft contact lenses |  |  |  |  |
| Hybrid contact lenses |  |  |  |  |
| RGP contact lenses |  |  |  |  |
| None |  |  |  |  |
| Refractive corrective aid – *Fellow eye* | n(%) |  |  |  |
| Glasses |  |  |  |  |
| Soft contact lenses |  |  |  |  |
| Hybrid contact lenses |  |  |  |  |
| RGP contact lenses |  |  |  |  |
| None |  |  |  |  |
| K_2_ (D) | mean(sd) |  |  |  |
| Study eye |  |  |  |  |
| Fellow eye |  |  |  |  |
| K_max_ (D) | mean(sd) |  |  |  |
| Study eye |  |  |  |  |
| Fellow eye |  |  |  |  |
| Apical corneal thickness (µm) | mean(sd) |  |  |  |
| Study eye |  |  |  |  |
| Fellow eye |  |  |  |  |
| Visual acuity (logMAR) | mean(sd) |  |  |  |
| Study eye |  |  |  |  |
| Fellow eye |  |  |  |  |
| Refraction (Spherical equivalent (D)) | mean (sd) |  |  |  |
| Study eye |  |  |  |  |
| Fellow eye |  |  |  |  |
| Refraction (Spherocylinder) | mean |  |  |  |
| Study eye |  |  |  |  |
| Fellow eye |  |  |  |  |
| Refractive astigmatism | n (%) |  |  |  |
| Study eye |  |  |  |  |
| Fellow eye |  |  |  |  |
| CHU9D | mean(sd) |  |  |  |
| CVAQC | mean(sd) |  |  |  |

**Table A2. Primary and Secondary outcome**

|  |  | **CXL** | **Standard Care** | **Adjusted coefficient**  **(95% CI)** | **p-value** |
| --- | --- | --- | --- | --- | --- |
| **Primary outcome** | | | | | |
| K_2_ (D) at 18 months ITT | mean(sd) |  |  |  |  |
| **Sensitivity Analysis of Primary outcome** | | | | | |
| K_2_ (D) at 18 months PP | mean(sd) |  |  |  |  |
| **Secondary outcome** | | | | | |
| Apical corneal thickness (µm) | mean(sd) |  |  |  |  |
| Visual acuity (logMAR) | mean(sd) |  |  |  |  |
| Refraction (Spherical equivalent (D)) | mean(sd) |  |  |  |  |
| CHU9D | mean(sd) |  |  |  |  |
| CVAQC | mean(sd) |  |  |  |  |
|  |  |  |  | **Adjusted odds ratio (95% CI)** |  |
| Keratoconus progression | n (%) |  |  |  |  |
| Refractive astigmatism | n (%) |  |  |  |  |
|  |  |  |  | **Adjusted hazard ratio (95% CI)** |  |
| Time to Keratoconus progression (months) | Med (IQR) |  |  |  |  |
|  |  |  |  | ***w*-statistic** |  |
| Refraction (Spherocylinder)^1^ | mean |  |  |  |  |

1. Unadjusted analysis comparing baseline refraction to that at 18 months post-randomisation.

ITT = Intention-to-treat, PP = Per-protocol

**Table A3. Adverse events and Serious Adverse events**

|  | **CXL** | **Standard Care** | **Total** |
| --- | --- | --- | --- |
| **Number of Patients reporting at least one SAE/ SAR/ SUSAR, n(%)** |  |  |  |
| **Number of Events**  SAE |  |  |  |
| SAR |  |  |  |
| SUSAR |  |  |  |
| Total |  |  |  |
| **Number of Patients reporting at least one AE, n(%)** |  |  |  |
| **Total number of AEs, n** |  |  |  |

**Table A4. Subgroup analyses**

|  |  | **CXL** | **Standard Care** | **Adjusted coefficient**  **(95% CI)** | **p-value** | **Interaction test p-value** |
| --- | --- | --- | --- | --- | --- | --- |
| Eyes progressed | One eye |  |  |  |  |  |
|  | Both eyes |  |  |  |  |  |
|  | Ineligible eye |  |  |  |  |  |
| Family history of Keratoconus | No |  |  |  |  |  |
|  | Yes |  |  |  |  |  |
| Ethnicity | White |  |  |  |  |  |
|  | Asian or Asian British |  |  |  |  |  |
|  | Other^1^ |  |  |  |  |  |
| Atopy | No |  |  |  |  |  |
|  | Yes |  |  |  |  |  |

1. Includes patients who identify themselves as - Black/ Black British, Mixed or Other ethnic groups
