# Supplementary material for: Corneal cross-linking versus standard care in children with keratoconus – a randomised, multicentre, observer-masked trial of efficacy and safety (KERALINK): a statistical analysis plan
Source: Trials. 2020 Jun 12;21:523. doi: 10.1186/s13063-020-04392-1 (PMC7291687; doi:10.1186/s13063-020-04392-1)
Supplement: Supplementary file 2 — Additional file 2. Guidelines for the Content of Statistical Analysis Plans in Clinical Trials Checklist (1) (recommended by the EQUATOR Network). [file 13063_2020_4392_MOESM2_ESM.docx]

**Additional file 2:** Guidelines for the Content of Statistical Analysis Plans in Clinical Trials Checklist (1) (recommended by the EQUATOR Network)

**Reference**

1. Gamble C, Krishan A, Stocken D, Lewis S, Juszczak E, Doré C, et al. Guidelines for the Content of Statistical Analysis Plans in Clinical Trials. JAMA. 2017;318(23):2337–43.
